# Radiobiological comparison of holmium-166 and yttrium-90 for hepatocellular carcinoma treatment *in vitro*

**DOI:** 10.1097/MNM.0000000000002190

**Published:** 2026-06-04

**Authors:** Justine Perrin, Carolline Ntihabose, Tijmen H. de Wolf, Duschka Kleijn, Erik de Blois, Mark Konijnenberg, Antonia Denkova, Julie Nonnekens

**Affiliations:** a Department of Molecular Genetics; b Department of Radiology and Nuclear Medicine, Erasmus MC Cancer Institute, Erasmus University Medical Center; c Department of Hospital Pharmacy, Erasmus University Medical Center, Rotterdam, The Netherlands; d Department of Radiation Science and Technology, Delft University of Technology, Delft, The Netherlands

**Keywords:** DNA damage, liver cancer, radiobiology, radioembolization

## Abstract

Radioembolization for hepatocellular carcinoma uses microspheres radiolabeled with yttrium-90 or holmium-166, yet their radiobiological effectiveness has not been directly compared, leaving radionuclide selection uninformed by biological considerations. We assessed the survival and DNA damage induction following 1.5 or 2.5 Gy of [^166^Ho]Ho-DOTA or [^90^Y]Y-DOTA delivered over 24 h on three human hepatocellular carcinoma cell lines. Both radionuclides led to a similar, dose-dependent reduction of survival in all cell lines, with a modest but nonsignificant trend towards increased toxicity for [^166^Ho]Ho-DOTA. Notably, [^166^Ho]Ho-DOTA induced fewer, but larger DNA damage foci at earlier time points during irradiation, suggesting subtle differences in damage complexity or repair kinetics. These findings suggest that when delivering the same absorbed dose over 24 h, [^166^Ho]Ho-DOTA and [^90^Y]Y-DOTA demonstrate comparable cytotoxicity *in vitro*. These findings imply that in clinical settings, additional factors such as total injected activity and microsphere composition and biodistribution likely exert greater influence on treatment efficacy than radionuclide choice alone.

## Background

Hepatocellular carcinoma (HCC) has a relative survival rate at 5 years of only 18% [1], and is often diagnosed at an advanced stage, limiting curative treatment options [2]. For eligible patients, treatment guidelines include transarterial radioembolization (TARE), which delivers radioactive microspheres to the tumor site via intra-arterial injection [3,4].

Despite its potential benefits, two phase III trials found no improvement in overall survival with TARE compared to standard treatment, although fewer side effects were observed [5,6]. These results may be explained by a limited understanding of radiobiology and dosimetry, which has likely contributed to suboptimal patient selection. Supporting this, the DOSISPHERE-01 phase II trial demonstrated that treatment adjustment based on personalized dosimetry significantly improved objective response rates compared to standard dosimetry [7].

Currently, two radionuclides are clinically available for TARE: yttrium-90 and holmium-166. Both are β^−^-emitting radionuclides with distinct physical properties. yttrium-90 has a half-life of 64.1 h and decay energy of 2.27 MeV (99.9%), whereas holmium-166 has a shorter half-life of 26.8 h and decay energies of 1.85 MeV (50.0%) and 1.77 MeV (48.7%).

To date, no study has directly compared their radiobiological efficacy in the context of TARE. To address this knowledge gap, we investigated the survival and DNA damage kinetics in three HCC cell lines treated *in vitro* with equivalent cumulative absorbed doses of either radionuclide.

## Methods

### *In vitro* irradiation

The three HCC cell lines PLC/PRF/5, Huh7, and Hep3b were cultured as described in Supplemental Material and Methods, Supplemental Digital Content 1, https://links.lww.com/NMC/A419. Cells were seeded in different cell culture plates depending on the assay at least 4 h before irradiation (Supplemental Table 1, Supplemental Digital Content 1, https://links.lww.com/NMC/A419). For X-ray exposure, cells were irradiated with either 1.5 or 2.5 Gy in an RS320 irradiator (195 kV, 10 mA, 1.5956 Gy/min). For treatment with TARE, see Supplemental Material and Methods, Supplemental Digital Content 1, https://links.lww.com/NMC/A419 for details on radiochemistry and dosimetry. At the end of the 24 h of irradiation, the cell culture medium was removed, wells were washed with PBS (Gibco), and fresh culture medium was added.

### Clonogenic survival assays

Each cell line was seeded in 6-well plates; 400 cells were seeded per well for PLC/PRF/5 and Huh7 cell lines, while 800 cells were seeded per well for Hep3b cells. Eleven days after irradiation, cell culture medium was removed, and each well was washed once with DPBS (Gibco) before colonies were fixed and stained with a mixture of 50% methanol (Honeywell), 43% distilled water, 7% acetic acid (Honeywell), and 0.1% brilliant blue (Sigma Aldrich). Colonies were counted using a GelCount (Animalab) automatic counter. The surviving fraction was calculated as a fraction of the untreated control, and a one-way ANOVA test was used to assess significant (*P* < 0.05) differences in survival. Each experiment was performed as three independent experiments with technical triplicates.

### DNA damage assessment

Complete descriptions of DNA double-strand break (DSB) assessment can be found in Supplemental material and methods, Supplemental Digital Content 1, https://links.lww.com/NMC/A419. Briefly, for fixed imaging, cells were seeded on glass coverslips, irradiated, and fixed with paraformaldehyde 2% (Sigma Aldrich) in PBS at 0, 8, 16, and 24 h during [^166^Ho]Ho-DOTA or [^90^Y]Y-DOTA exposure. After permeabilization and blocking, cells were stained for 1 h at room temperature with anti-human 53BP1 (Bethyl A300-272A, polyclonal, Lot #7) and anti-human γH2AX (Merc Millipore 05-636, clone JBW301), diluted 1 : 500 in blocking buffer. After washing, cells were incubated for 1 h at room temperature with secondary anti-mouse Al488 (Invitrogen A21202) and anti-rabbit Al594 (Invitrogen A21207), diluted 1 : 1000 in blocking buffer. The coverslip was then mounted on a microscope slide (Epredia) with Vectashield mounting reagent containing DAPI (Vector Laboratories), and images were acquired on a SP8 Leica confocal microscope. For each condition, experiments were performed in biological triplicates, with at least 50 cells analyzed in total.

For live imaging, PLC/PRF/5_53BP1-mClover cells (see Supplemental Material and Methods, Supplemental Digital Content 1, https://links.lww.com/NMC/A419 for the generation and validation of the cell line) were seeded in an 8-well ibidi plate (Ibidi) and imaged with a SP8 Leica confocal microscope during 24 h while being irradiated with either [^166^Ho]Ho-DOTA or [^90^Y]Y-DOTA (Supplemental Figure 3, Supplemental Digital Content 1, https://links.lww.com/NMC/A419). Image analysis was done using Cellpose [8] for nuclei detection and Ilastik [9] for foci detection, using pre-existing methodology [10]. For each condition, experiments were performed in biological triplicates, with at least 50 cells analyzed in total.

## Results

### [^166^Ho]Ho-DOTA and [^90^Y]Y-DOTA induce comparable survival

Since proliferation speed can influence radiation sensitivity [11,12], the doubling times of PLC/PRF/5, Huh7 and Hep3b cell lines were first evaluated. These were 26.5, 28, and 25.5 h, respectively (Supplemental Figure 1, Supplemental Digital Content 1, https://links.lww.com/NMC/A419). The impact of the non-radioactive Ho-DOTA or Y-DOTA, applied at concentrations equivalent to those of the radioactive counterparts, was assessed, and no significant effect on cell survival was observed in any of the cell lines (Supplemental Figure 2, Supplemental Digital Content 1, https://links.lww.com/NMC/A419).

Treatment doses were chosen based on the survival fraction of approximately 50% induced by X-rays in all three cell lines at 1.5 or 2.5 Gy. Treatment with 1.5 Gy delivered with X-rays, [^166^Ho]Ho-DOTA, or [^90^Y]Y-DOTA induced a reduction in survival across all cell lines. No significant difference in cell killing capacity was observed between the different irradiation types for PLC/PRF/5 and Hep3b cells (Fig. 1a and c). However, Huh7 cells exhibited significantly greater sensitivity to X-rays compared to [^166^Ho]Ho-DOTA or [^90^Y]Y-DOTA.

**Fig. 1:**
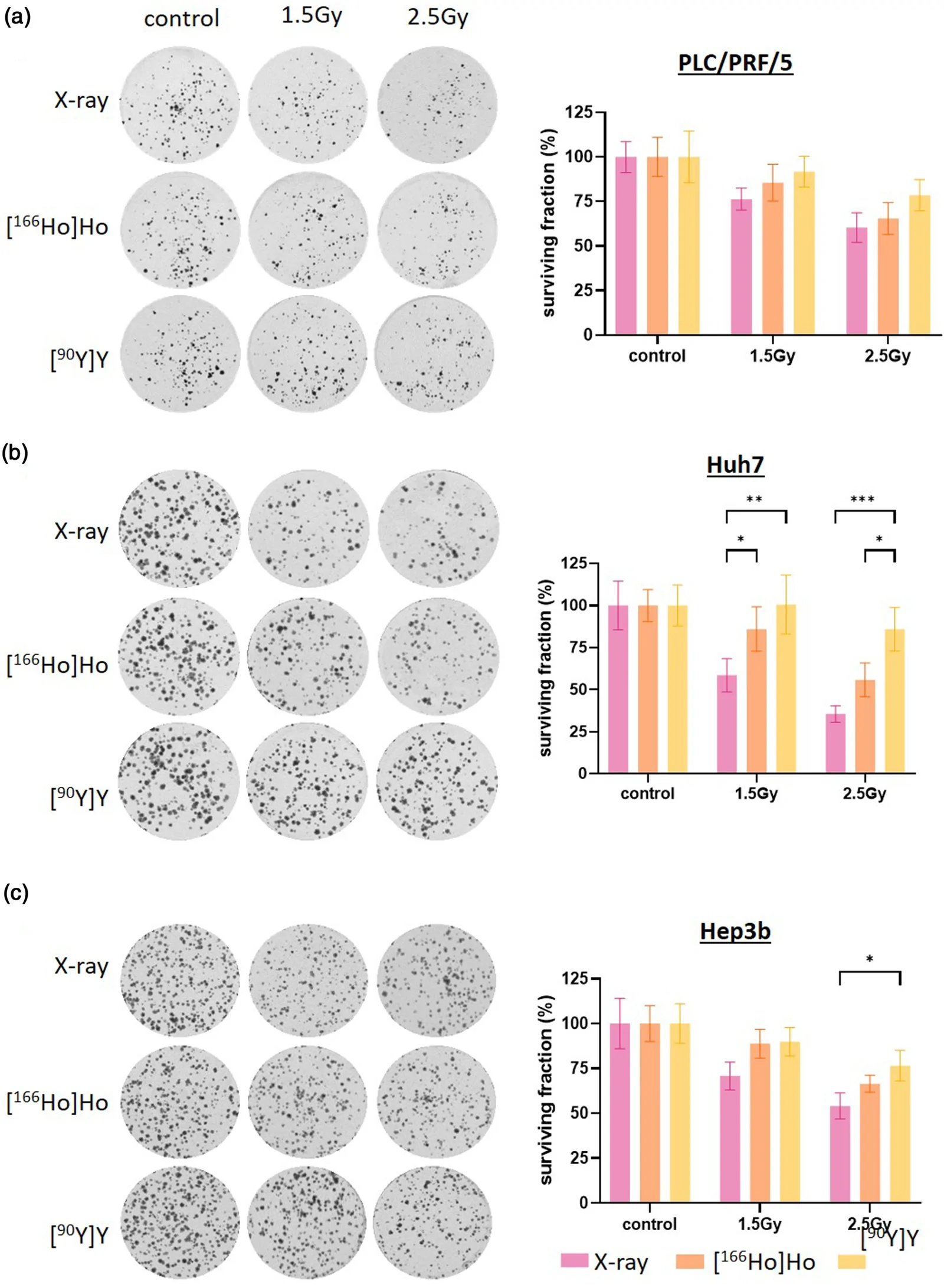
Survival of (a) PLC/PRF/5, (b) Huh7, (c) Hep3b cells after incubation with 1.5 or 2.5 Gy of X-ray, [^166^Ho]Ho-DOTA, or [^90^Y]Y-DOTA compared to unirradiated control. Left panels: representative images of clonogenic survival assays. Right panels: quantification of survival fraction (*n* = 3 independent experiments, mean ± SD *P < 0.05, **P < 0.005, ***P < 0.0005, ANOVA test).

At 2.5 Gy, a further dose-dependent decrease in survival was observed for all cell lines and radiation types. Interestingly, Huh7 cells showed a significant increase in cell killing with [^166^Ho]Ho-DOTA compared to [^90^Y]Y-DOTA. The other cell lines and treatment conditions showed a similar, though non-significant, trend towards higher cytotoxicity for [^166^Ho]Ho-DOTA.

### Dose-dependent induction of 53BP1 and γH2AX foci across hepatocellular carcinoma cell lines

The level of DSB induction was visualized by quantifying the number of 53BP1 and γH2AX foci in each cell line during the 24 h of irradiation with 1.5 or 2.5 Gy of [^166^Ho]Ho-DOTA or [^90^Y]Y-DOTA (Fig. 2). A significant, dose-dependent increase in both 53BP1 and γH2AX foci numbers was observed in all three cell lines over time (*P* < 0.05). On average, the highest number of foci per nucleus was reached at 8 hours after the start of irradiation, after which a plateau was observed.

**Fig. 2:**
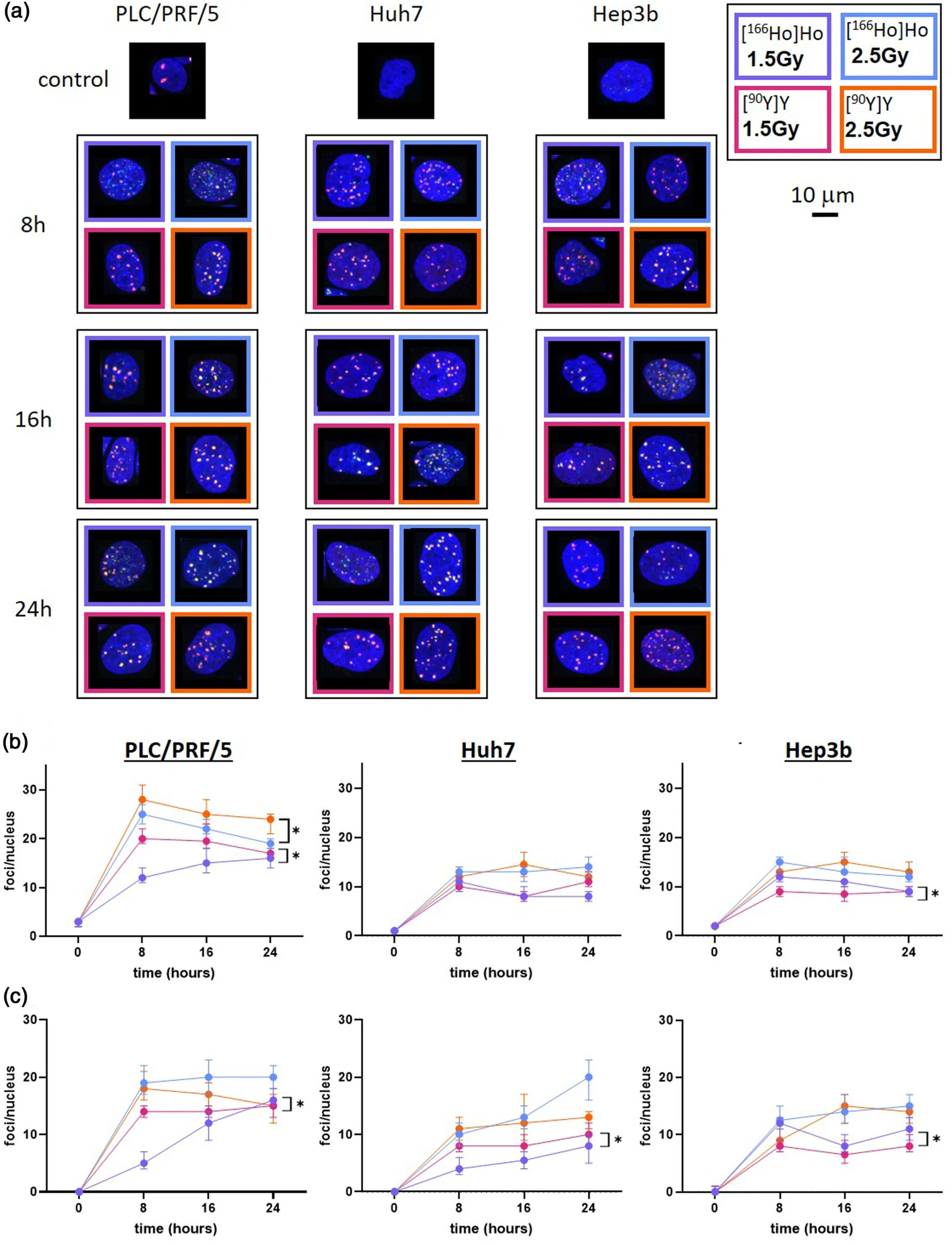
(a) Representative nucleus (blue: DAPI, red: 53BP1, γH2AX: green), and quantification of the number of (b) 53BP1 and (c) γH2AX foci per nucleus in each cell line during treatment with either 1.5 or 2.5 Gy of [^166^Ho]Ho-DOTA or [^90^Y]Y-DOTA (*n* = 3 independent experiments, mean ± CI, *P < 0.05, ANOVA test).

The PLC/PRF/5 cells showed a significantly higher number of both 53BP1 and γH2AX foci per nucleus when treated with 1.5 Gy of [^90^Y]Y-DOTA compared to 1.5 Gy of [^166^Ho]Ho-DOTA (*P* < 0.005). A similar trend was observed at 2.5 Gy, but significance was reached only for 53BP1 and not γH2AX (*P* < 0.005). Huh7 cells exhibited a similar trend, showing a significantly higher number of γH2AX foci when irradiated with 1.5 Gy of [^90^Y]Y-DOTA compared to [^166^Ho]Ho-DOTA (*P* < 0.005). In contrast, Hep3B cells showed a significantly higher induction of both 53BP1 and γH2AX foci when treated with 1.5 Gy of [^166^Ho]Ho-DOTA compared to [^90^Y]Y-DOTA (*P* < 0.005).

### [166Ho]Ho-DOTA induces larger foci than [90Y]Y-DOTA at early time points

Consistent with fixed-cell imaging of the PLC/PRF/5 wild-type cells, [^90^Y]Y-DOTA induced a higher number of foci per nucleus than [^166^Ho]Ho-DOTA (Fig. 3a and b). Irradiation with [^90^Y]Y-DOTA resulted in a rapid induction of foci, reaching a plateau within the first hour, whereas irradiation with [^166^Ho]Ho-DOTA led to a more gradual increase in foci formation over the 24-h period.

**Fig. 3:**
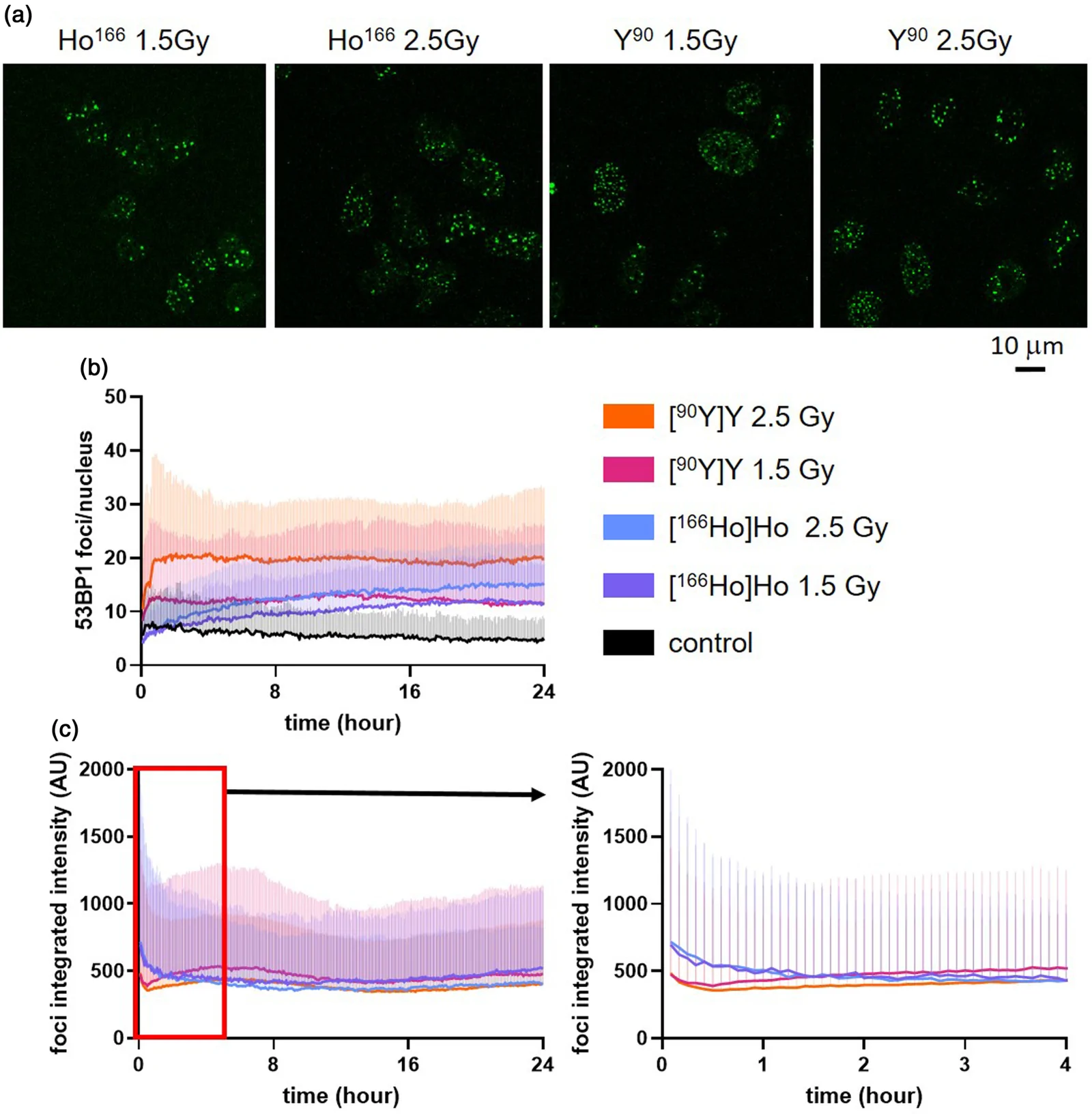
(a) Representative screenshots of irradiated cells for the 1.5 and 2.5 Gy irradiation with either [^166^Ho]Ho-DOTA or [^90^Y]Y-DOTA. Quantification of the (b) number of 53BP1 foci per nucleus and (c) foci integrated intensity in the PLC/PRF/5 53BP1mClover cell line during irradiation with either 1.5 or 2.5 Gy of [^166^Ho]Ho-DOTA or [^90^Y]Y-DOTA (*n* = 3 independent experiments, mean ± SD).

Foci size analysis showed that [^166^Ho]Ho-DOTA induced larger foci compared to [^90^Y]Y-DOTA at early time points, independent of the dose, although this difference was NS (Fig. 3c).

## Discussion

Although previous meta-analyses of TARE suggested a threshold dose of 100–250 Gy to induce tumor control, highlighting the importance of delivered dose [13], standardized dosimetry approaches and clear guidance on radionuclide selection are still lacking [14]. To compare the impact of holmium-166 and yttrium-90 on HCC cell killing, HCC cell lines were irradiated *in vitro* under controlled dosimetry conditions. Clonogenic survival assays showed a slight trend towards greater cytotoxicity with [^166^Ho]Ho-DOTA compared to [^90^Y]Y-DOTA; however, this difference was only significant in Huh7 cells at 2.5 Gy. These findings suggest that the delivery of the same cumulative absorbed dose over 24 h results in largely comparable cell killing efficacy between the two radionuclides.

Interestingly, DSB levels did not directly align with survival trends. [^90^Y]Y-DOTA induced more DSBs per cell than [^166^Ho]Ho-DOTA in PLC/PRF/5 and Huh7 cells, while the opposite was observed in Hep3b cells. Live-cell imaging of 53BP1-mClover-expressing PLC/PRF/5 cells showed that [^90^Y]Y-DOTA induced slightly higher DSB numbers, yet [^166^Ho]Ho-DOTA produced larger foci at early time points. This may indicate the induction of more complex DSBs requiring greater 53BP1 recruitment for repair, potentially linked to the higher initial dose rate of [^166^Ho]Ho-DOTA relative to [^90^Y]Y-DOTA. Such damage complexity could contribute to the modest trend towards lower survival following [^166^Ho]Ho-DOTA treatment.

Compared with clinical TARE, the *in vitro* settings used here involved substantially lower cumulative absorbed doses (maximum of 2.5 Gy over 24 h), whereas clinical treatments typically deliver 100–250 Gy over a longer time period. Under our controlled experimental conditions, only minor differences in HCC cell killing and DSB induction were observed between [^166^Ho]Ho-DOTA and [^90^Y]Y-DOTA. This outcome is supported by the fact that, although holmium-166 and yttrium-90 differ in half‑life and emission spectra, their overall physical characteristics are sufficiently similar that major differences in radiobiological effectiveness would not be anticipated, which is consistent with the absence of such differences in our experiments. Within this context, the comparable radiobiological effects observed do not support defining specific clinical scenarios favoring one radionuclide over the other based on radiobiology alone. Taken together, these findings imply that, in clinical practice, parameters such as total injected activity, microsphere composition and distribution, as well as practical and logistical considerations (e.g., availability, regulatory aspects, and imaging capabilities), are likely to have a greater impact on treatment efficacy than the choice between holmium-166 and yttrium-90 itself.

## Acknowledgements

The authors would like to thank Baukje Terpstra and the TU Delft Reactor Institute for the irradiations of [^166^Ho]Ho and [^90^Y]Y and the Erasmus MC Optical Imaging Center for their support in live cell imaging. Figures were created with BioRender.com.

This study was funded by Quirem Medical, a Terumo company and by the Erasmus MC, Fellowship project RADIANT.

The datasets and materials created during the current study are available from the corresponding author on reasonable request.

Data have previously been presented at the Annual Congress of the European Association of Nuclear Medicine, Sept 2024 in Hamburg, Germany. The abstract is published as: Perrin J, Ntihabose C, Zink J, de Blois E, Konijnenberg M, Denkova A, Nonnekens J. Radiobiological comparison of yttrium-90 and holmium-166 in liver cancer cells. Eur. J. Nucl. Med. Volume 51, Page S129-S129, supplement: 1, Meeting abstract OP-276.

### Conflicts of interest

J.N. has received research funding from Quirem Medical, a Terumo Company. For the remaining authors, there are no conflicts of interest.

## Supplementary Material
