# Supplementary material for: Radiobiological comparison of holmium-166 and yttrium-90 for hepatocellular carcinoma treatment in vitro
Source: Nucl Med Commun. 2026 Jun 4;47(11):1274–80. doi: 10.1097/MNM.0000000000002190 (PMC13641249; doi:10.1097/MNM.0000000000002190)
Supplement: Supplementary file 1 [file nmc-47-1274-s001.docx]

**Supplemental Tables and Figures**

**Supplemental Table 1**: **Experiment setup.** Activities, cell culture plates and corresponding volumes used to deliver 1.5 or 2.5 Gy of [^166^Ho]Ho or [^90^Y]Y in each experiment.

|  |  | [^166^Ho]Ho-DOTA | | [^90^Y]Y-DOTA | |
| --- | --- | --- | --- | --- | --- |
|  |  | 1.5Gy | 2.5Gy | 1.5Gy | 2.5Gy |
| Clonogenic survival | type of plate and volume | 6 well (1.5mL) | 6 well (1.5mL) | 6 well (1.5mL) | 6 well (1.5mL) |
|  | corresponding activity (MBq) | **0.825** | **1.374** | **0.646** | **1.077** |
| DNA damage (fixed) | type of plate and volume | 24 well (0.75mL) | 24 well (0.75mL) | 24 well (0.75mL) | 24 well (0.75mL) |
|  | corresponding activity (MBq) | **0.445** | **0.742** | **0.323** | **0.538** |
| DNA damage (live imaging) | type of plate and volume | 8 well ibidi (0.3mL) | 8 well ibidi (0.3mL) | 8 well ibidi (0.3mL) | 8 well ibidi (0.3mL) |
|  | corresponding activity (MBq) | **0.159** | **0.265** | **0.121** | **0.201** |

**a)**

**b)**

**c)**

**Supplemental Figure 1: HCC cells growth curves.** Number of cells for the a) PLC/PRF/5 b) Huh7 and c) Hep3b cell line across time.

**Supplemental Figure 2: Survival of HCC cells after 24 hours incubation with the non-radioactive Ho-DOTA or Y-DOTA**. a) Representative images of clonogenic survival assays and b) quantification of the survival fraction compared to the control of each cell line (n=3 independent experiments, mean +/- SD).

**Supplemental Figure 3: Validation of the PLC/PRF/5_53BP1-mClover clones.** a) PCR strategy for amplification of the inserted mClover gene. b) PCR amplification product after migration in an agarose gel. PCR was conducted in duplicate for all PLC/PRF/5 clones (2C5, 3C3, 1G5, 1G6, 1D5) and Wild Type (WT, right) c) Clonogenic survival of PLC/PRF/5_53BP1-mClover transfected clones compared to the WT cell line after treatment with 0, 1, 2 or 2.5 Gy (n=2). d) Representative maximum projection of the PLC/PRF/5_53BP1-mClover 1D5 clone 53BP1 staining and e) 53BP1-mClover intrinsic signal Quantification of 53BP1 foci per nucleus in each clone after 1Gy of X-ray across 24 hours.

**Supplemental material and methods**

**Cell lines**

The three HCC cell lines PLC/PRF/5, Huh7 and Hep3b 1 were a kind gift from Dr. Ron Smits (Erasmus MC, department of Gastroenterology & Hepatology). Cells were maintained at 37°C, 5% CO2 in a humid atmosphere, in DMEM high glucose (Gibco) supplemented with 1% penicillin-streptomycin (Gibco) and 10% Fetal Bovine Serum (Serana). Each cell line’s doubling time was calculated by plating 50000 cells in a 6-well plate and counting them with the Countess 3 Automated Cell Counter (AMQAX2000, ThermoFisher Scientific) after dilution 1:1 in trypan blue. Cells were counted every 24 hours for 4 consecutive days and an exponential growth curve was fitted to determine the doubling times.

**Dosimetry**

For the modeling of the irradiation with [^166^Ho]Ho-DOTA or [^90^Y]Y-DOTA (see labeling material and methods in the Supplementals), cells were considered as a 10 μm layer at the bottom of the well. Dosimetry calculations were based on the cell culture well volume to deliver, over 24 hours, 1.5 or 2.5 Gy to the cells using MCNP6.2 code (MCNP User's Manual Code Version 6.2, Los Alamos National Laboratory Tech. Rep. LA-UR-17-29981), as previously described ^2^.

**Radiochemistry**

Holmium(III) nitrate pentahydrate (99.9% trace metal basis) and Yttrium (500μm maximum particle. size purity 99.9%) powder were purchased from Merck and were irradiated at the Hogert Onderwijs Reactor of the TU Delft, the Netherlands. The pneumatic irradiation facility was used for the irradiation, having the following thermal and epithermal neutron flux respectively: 3.11×10^16^ n/(s^-1^m^2^) and 7.2×10^14^n/(s^-1^m^2^). The irradiations were carried out for varying durations to achieve the desired activity. The activities of both radionuclides were measured using a dose calibrator (VIK-202 and VDC 404). [^166^Ho]Ho was dissolved in 100 µL of 0.2 M HCl, [^90^Y]Y was dissolved in 120 µL of pure HCl (12.18 M) and thoroughly mixed using a vortex. The solution was subsequently transferred to a 1.5 mL microtube vial. 3.7 mg and 1.5mg of 1,4,7,10-Tetraazacyclododecane-1,4,7,^10^-tetraacetic acid (DOTA, Sigma-Aldrich) was added to the [^166^Ho]Ho or [^90^Y]Y solution, respectively. The pH of each solution was adjusted to 4 with 1 M NaOH and 4.1 M HCl, the solution was vortexed and incubated at 90°C for 1 hour to ensure chelation.

30-35 mg of Ln-Resin-B (Triskem LN-B25-S) was weighed and packed into CHROMABOND columns Shorty PS-HCO3 (Macherey Nagel 730691). The resin was pre-rinsed with LC-MS grade water, followed by the incubation step. The chelated [^166^Ho]Ho-DOTA or [^90^Y]Y-DOTA solutions were diluted to a total volume of 1 mL using LC-MS grade water and passed through the prepared resin columns to trap the unchelated nuclides. The columns were washed with additional water, and the eluate was collected in 1.5 mL microtube vials. Purity of the isolated chelated [^166^Ho]Ho-DOTA or [^90^Y]Y-DOTA was verified by measuring the radioactivity retained on the column versus the eluate using a dose calibrator (VIK-202 and VDC 404) and additionally further confirmed using an automated gamma counter (2480 Wizard-2, PerkinElmer), which was set to the open-channel and calibrated for appropriate energy settings. For control experiments, identical chelation of the non-radioactive Holmium(III) nitrate pentahydrate and Yttrium powder were done.

**DNA damage assessment – fixed imaging**

The number of DNA double strand breaks (DSBs) was measured by quantification of immunofluorescent staining of 53BP1 and γH2AX foci per nucleus. 100 000 cells were seeded on coverslips in 24-well plates 6h before irradiation, and at 0, 8, 16 and 24 hours during [^166^Ho]Ho-DOTA or [^90^Y]Y-DOTA exposure, cell culture medium was removed. Cells were washed once with DPBS (Gibco), fixed using paraformaldehyde 2% (Sigma Aldrich) and permeabilized with 0.1% triton (Sigma Aldrich) diluted in DPBS (Gibco). Non-specific binding sites were blocked by washing cells with blocking buffer: DPBS 0.1% triton 1% bovine serum albumin (Sigma Aldrich). Cells were stained for 1 hour at room temperature with anti-human 53BP1 (Bethyl A300-272A, polyclonal, Lot #7) and anti-human γH2AX (Merc Millipore 05-636, clone JBW301), diluted 1:500 in blocking buffer. After washing five times with blocking buffer, cells were incubated for 1 hour at room temperature with secondary antibodies anti-mouse Al488 (Invitrogen A21202) and anti-rabbit Al594 (Invitrogen A21207), diluted 1:1000 blocking buffer. The coverslip was mounted on a microscope slide (Epredia) with Vectashield mounting reagent containing DAPI (Vector laboratories) and images were acquired on a SP8 Leica confocal microscope using a 63x immersion objective, with a resolution of 1024 x 1024 pixels. The number, size and intensity of 53BP1 and γH2AX foci were quantified using a custom-made ImageJ macro. For each condition, 3 fields of view with a z-stack using a step of 1mm were acquired with at least 6 cells per field of view, and each experiment was performed as three independent replicates. 2-way ANOVA test was used to assess significant (p<0.05) difference in DNA damage induction between conditions.

**53BP1-mClover expression**

To perform live cell imaging, endogenous fluorescently-tagged 53BP1 cells were created. For this, PLC/PRF/5 cells were transfected with 1 µg of a donor plasmid containing the mClover sequence flanked by 53BP1 homologous sequence, 1 µg of guide plasmid encoding the CRISPR-Cas9 sequence, and the guide RNA targeting the 53BP1 locus using X-treme gene transfection reagent (Sigma Aldrich) according to the manufacturer’s protocol. Transfected cells were selected with 1.25 μg/mL puromycin (Invitrogen) and mClover-positive cells were bulk sorted by flow cytometry (LSR Fortessa X-20). Single clones were isolated by limiting dilution: cells were plated at an average of 0.5 cells/well in a 96-well plate and positive 53BP1-mClover clones were validated by immunofluorescence staining and genomic PCR followed by agarose gel analysis (forward primer: ggtgatccagtgcctcattgttgg, reverse primer: gctgtcctccgtaagtgaataagcc).

**DNA damage assessment – live imaging**

For each acquisition, 9000 PLC/PRF/5_53BP1-mClover cells (see generation of the cell line material and methods in Supplemental) were seeded per well in an 8-well ibidi plate (ibidi) 24 hours before imaging with a SP8 Leica confocal microscope, a 40x immersion objective and an image resolution of 1024 x 1024 pixels. Cells were kept at 37°C, 5% CO2 and humid atmosphere during 24 hours of imaging while being irradiated with either [^166^Ho]Ho-DOTA or [^90^Y]Y-DOTA. For each condition, 4 fields of view were imaged per experiment with time interval of 5 minutes, with a z-stack using a step of 0.75 mm. Image analysis was done using Cellpose ^3^ for nuclei detection and ilastik ^4^ for foci detection, using pre-existing methodology ^5^. The number of foci per nucleus was counted and the integrated intensity was calculated by multiplying the size per intensity of each focus. For each condition, 4 fields of view with on average 12 cells were acquired, and experiments were performed in three independent replicates.

**References**

1.	He L, Tian DA, Li PY, He XX. Mouse models of liver cancer: Progress and recommendations. *Oncotarget*. 2015;6(27):23306-23322. doi:10.18632/oncotarget.4202

2.	Chan HS, de Blois E, Morgenstern A, et al. In Vitro comparison of 213Bi- and 177Lu-radiation for peptide receptor radionuclide therapy. Xu B, ed. *PLoS One*. 2017;12(7):e0181473. doi:10.1371/journal.pone.0181473

3.	Stringer C, Wang T, Michaelos M, Pachitariu M. Cellpose: a generalist algorithm for cellular segmentation. *Nat Methods*. 2021;18(1):100-106. doi:10.1038/s41592-020-01018-x

4.	Berg S, Kutra D, Kroeger T, et al. ilastik: interactive machine learning for (bio)image analysis. *Nat Methods*. 2019;16(12):1226-1232. doi:10.1038/s41592-019-0582-9

5.	Stringer C. Cellpose. Published 2025. https://github.com/MouseLand/cellpose?tab=readme-ov-file
